# Electrospun Biomimetic Nanofibrous Scaffolds: A Promising Prospect for Bone Tissue Engineering and Regenerative Medicine

**DOI:** 10.3390/ijms23169206

**Published:** 2022-08-16

**Authors:** Shabnam Anjum, Farheen Rahman, Prashant Pandey, Dilip Kumar Arya, Mahmood Alam, Paruvathanahalli Siddalingam Rajinikanth, Qiang Ao

**Affiliations:** 1 Department of Tissue Engineering, School of Intelligent Medicine, China Medical University, Shenyang 110122, China; 2 Department of Applied Chemistry, Zakir Husain College of Engineering & Technology, Aligarh Muslim University, Aligarh 202002, India; 3 Department of Pharmaceutical Sciences, Babasaheb Bhimrao Ambedkar University, Vidya Vihar, Raebareli Road, Lucknow 226025, India; 4 Department of Clinical Medicine, China Medical University, Shenyang 110122, China; 5 NMPA Key Laboratory for Quality Research and Control of Tissue Regenerative Biomaterial & Institute of Regulatory Science for Medical Device & National Engineering Research Center for Biomaterials, Sichuan University, Chengdu 610064, China

**Keywords:** bone tissue regeneration, nanofibrous scaffolds, electrospinning, nanofiber composite, bone defects, growth factor

## Abstract

Skeletal-related disorders such as arthritis, bone cancer, osteosarcoma, and osteoarthritis are among the most common reasons for mortality in humans at present. Nanostructured scaffolds have been discovered to be more efficient for bone regeneration than macro/micro-sized scaffolds because they sufficiently permit cell adhesion, proliferation, and chemical transformation. Nanofibrous scaffolds mimicking artificial extracellular matrices provide a natural environment for tissue regeneration owing to their large surface area, high porosity, and appreciable drug loading capacity. Here, we review recent progress and possible future prospective electrospun nanofibrous scaffolds for bone tissue engineering. Electrospun nanofibrous scaffolds have demonstrated promising potential in bone tissue regeneration using a variety of nanomaterials. This review focused on the crucial role of electrospun nanofibrous scaffolds in biological applications, including drug/growth factor delivery to bone tissue regeneration. Natural and synthetic polymeric nanofibrous scaffolds are extensively inspected to regenerate bone tissue. We focused mainly on the significant impact of nanofibrous composite scaffolds on cell adhesion and function, and different composites of organic/inorganic nanoparticles with nanofiber scaffolds. This analysis provides an overview of nanofibrous scaffold-based bone regeneration strategies; however, the same concepts can be applied to other organ and tissue regeneration tactics.

## 1. Introduction

### 1.1. Bone Defect—Challenges and Current Treatment

Since the advent of living species until now, different kinds of disorders, diseases, and infections have been faced by human beings. Among them, bone-related disorders have a major influence on the population worldwide. The reconstruction and healing of body tissues and organs are substantial obstacles for physicians and tissue engineers. The increased incidence of tissue injury and organ damage has increased the requirement for organ transplantation year after year. Bone is the most often transplanted tissue after blood [1]. Many studies revealed that bone regeneration is one of the most complicated issues. Notably, bone defects or dysfunction affect approximately 20 million people annually related to different kinds of bone-related disorders, namely, congenital anomalies, trauma, or another disease [1,2]. Bone grafts or bone replacements are used in an estimated 2.2 million surgeries annually [3,4].

Bone serves various functions, including primary backing structure, binding position for tendons, ligaments and muscles, mechanical strength, and protection of vital tissues and organs. The bone marrow structure also provides hematopoiesis and essential mineral materials [5,6]. Bone-related diseases such as osteoporosis, bone cancer, and bone infection have become a significant concern for modern communities as the prevalence of these diseases has increased [7]. Most of the bone fractures are caused by inconvenient or ineffective bone tissue restoration. While bone tissue has an excellent capability for regeneration, significant bone deficiencies caused by tumor removal, cancer, contagious illness, extreme mechanical damage, and inherited abnormalities do not heal on their own and necessitate extensive bone grafting [8]. In orthopedic and reconstructive surgery, bone is one of the most often transplanted tissues.

**Figure 1:**
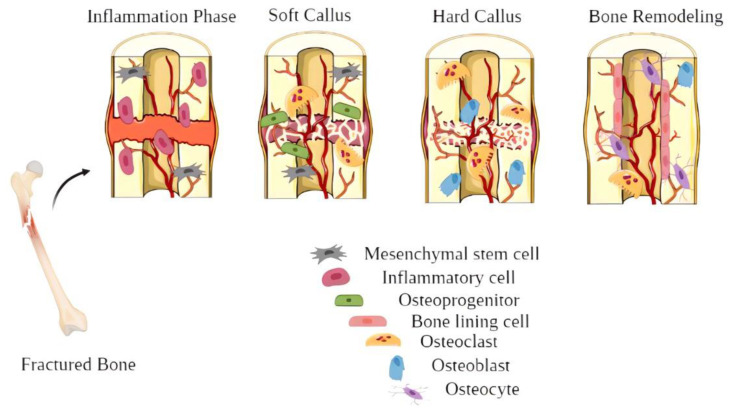
Illustration of the bone healing process.

Bone fracture regeneration is a multistep, organized process involving many progenitor cells and endothelial, inflammatory and hematopoietic cells [9]. Osteoprogenitor cells are the primary bone cells that can differentiate into mature bone cells and can be activated and converted into osteoblasts during bone damage and repair. Proliferation, matrix maturation and mineralization are the three stages of osteogenic differentiation that permit osteoblasts to develop new bones [10,11]. The bone regeneration process involves three steps: inflammation, bone development, and bone remodeling (Figure 1) [12]. When a bone is fractured, it starts bleeding into the surrounding area, which causes inflammation and blood clotting at the fracture site. This is the primary source of fundamental strength and the foundation for new bone growth. Bone formation begins when inflammation-induced blood clots are replaced by stringy tissue and cartilage (acknowledged as a soft callus). Hard bone (known as a hard callus) replaces the soft callus once it regenerates, which can be seen on X-rays a few weeks after the injury. Bone remodeling, the last step of bone repair, will take months to complete [13,14].

Current medical treatment approaches to bone repair comprise allogeneic (grafts derived from some other member of the same species; normally cadaveric bone collected from a bone bank) or autologous (transfer of bone grafts from a donor to a recipient located in the same patient) bone grafts. Autologous bone grafting remains the gold standard due to its optimal osteoconductive, osteoinductive, and osteogenic properties. On the other hand, these replacements have certain disadvantages, such as the chance of immunological failure and the transfer of pathogen through source towards the recipient, donor scarcity, and high cost [15,16]. For these issues, a better effective clinical therapy strategy is needed. Tissue engineering is a cutting-edge medical technique that has produced remarkable achievements in reducing the risks associated with numerous surgical procedures. Therefore, bone tissue engineering requires therapeutic treatment for damaged bones and eliminates the risk of donor inadequacy, supply constraints, and immunological refusal [17].

Bone tissue engineering (BTE) procedures make usage of various scaffolds (e.g., composite scaffolds, nanofibrous scaffolds, porous scaffolds, hydrogel scaffolds) in conjunction with biological materials. The development of a bio-artificial bone graft, a scaffold that replicates extracellular matrix (ECM) while also having osteoconductive functionality that may be used to restore broken or damaged bones, is a major problem in BTE. To address the demand for successful bone tissue regeneration (BTR), a method for developing biomaterials, including nanosized topographical design, micro- and macro-scale graded geometries and biological arenas that combine with specific growth factors (GFs) and cells, is required [18,19]. The intrinsic porosity of the bone tissue scaffold is an important characteristic that permits vast numbers of osteogenic cells to integrate and develop tissues. A structure having significant porosity, linked pores, and a wide surface area has been shown to promote tissue in-growth [20]. Scaffolds can stimulate bone regeneration and disintegrate over time or provide a lifelong substitute, which is the more advanced measure to bridge the bone defect as an alternative to traditional methods of bone grafting. Besides natural and artificial bone grafting for BTR, modern surgical methods also employ drug/GF-loaded scaffolds. To augment the therapeutic efficiency of bone regeneration, several biodegradable natural and artificial polymer-based drug delivery arrangements have been developed and tested in in vivo and in vitro studies [21,22].

One of the critical problems in BTE is the creation of scaffolds with outstanding mechanical characteristics, biodegradability, and designs for cell growth and migration that might allow the scaffold’s interface with the host tissue. Bone tissue engineers are now exploring several options in this area, formulating novel structures using a comprehensive set of biomaterials and cell therapy to boost bone and joint health. Once inside the body, ideal scaffolds should retain adequate mechanical properties, degrade in a regulated manner without disarranging toxic materials, regulate the release of loaded drugs or biomolecules in a time and space-controlled manner, and direct cell activity to replicate the hierarchical architecture of primitive bone tissue.

**Figure 2:**
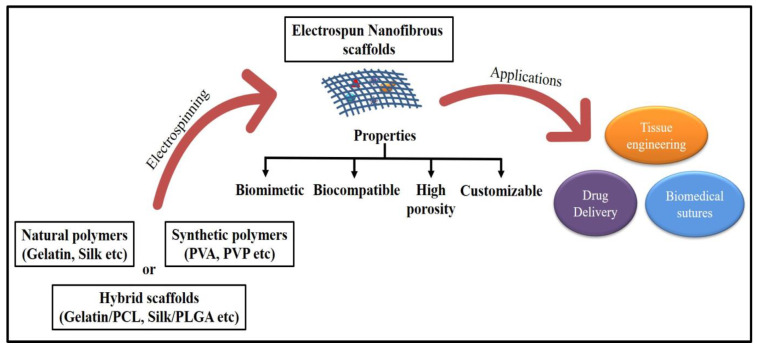
Electrospun nanofibrous scaffolds for biomedical applications.

The electrospinning technique to create these scaffolds has gained tremendous interest because the nanofibers are biomimetic of the ECM in natural bone, which entails mainly hierarchically structured, mineralized collagen fibers, as well as simple setup and operation at low cost [23]. The electrospinning process is appealing for tissue engineering, drug delivery, and other biomedical uses due to its ease of use and high surface-to-volume ratio (Figure 2). Nanostructured particles, including nanofibrous scaffolds, have been extensively researched to improve the current treatment method’s effectiveness and specificity.

The latest progress made in the field of electrospun nanofibrous scaffolds for BTR is summarized in Table 1.

### 1.2. Biology of Bones

**Figure 3:**
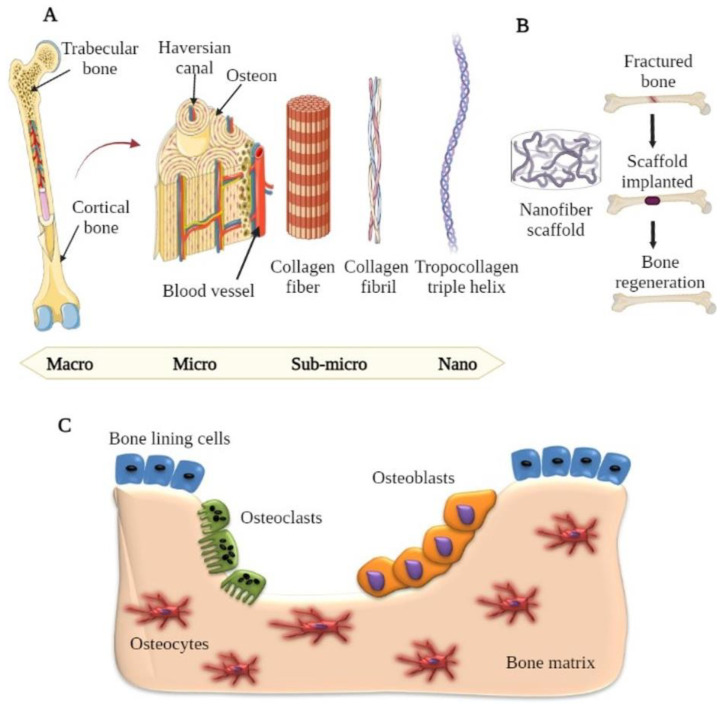
(**A**) Hierarchical structure of natural bone tissue from macro to nano scale. (**B**) Cumulative illustration of bone tissue engineering using nanofiber scaffold. (**C**) Representation of different types of bone cells required for bone regeneration.

Bones comprise a group of tissues in the human body with a complex hierarchically arranged structure from the nano (collagen and hydroxyapatite) to the macro (cancellous and cortical bone) scale (Figure 3) [30,31]. The hard outer layer of bone is cortical bone, which consists of Haversian canals and osteons, while the inside structure (spongy bone) has a trabecular arrangement with a porosity of 75–85% [32]. The surface of bones consists of a complicated arrangement of parallel type I collagen nanofibrils and hydroxyapatite crystals. The bone matrix comprises approximately 10% water, 25% organic, and 65% mineral components. Collagen type I (90%) and type V (in minor amounts) are the most abundant proteins in the organic matrix, with non-collagenous proteins including phospholipids, osteonectin, osteocalcin, osteopontin, and fibronectin contributing to the remaining 10% [33,34]. The mechanical strength and tissue adhesive properties of bone matrix proteins are crucial. With bicarbonates, citrates, and ions such as F^−^, Pb^2+^, Zn^2+^, Cu^2+^, Fe^2+^, K^+^, and Sr^2+^, hydroxyapatite (HA) [Ca_10_(PO_4_)_6_(OH)_2_] is the most essential element of the inorganic mineral phase of bone [35,36].

The basic multicellular unit (BMU) comprises the four primary essential cells involved in bone structure and regeneration: osteogenic, osteocytes, osteoblasts, and osteoclasts [37,38]. Osteoblasts are mesenchymal-derived osteoprogenitor cells in the bone marrow and other connective tissues. Osteoblasts secrete type I collagen and a variety of non-collagenous proteins, including osteopontin, osteocalcin, phospholipids, and bone sialoprotein, during the ossification phase. These cells are in control of bone growth and remodeling [10,38,39]. Osteocytes are the most prevalent cell type found in bone tissue. MSCs (mesenchymal stem cells) that have undergone osteoblastic differentiation produce osteocytes. These osteoblasts are no longer active and become stuck in the bone they formed. They keep in touch with other osteoblasts and osteocytes. They are required for the interaction of bone tissue. Moreover, osteocytes were found to interact with various bio-chemical signaling pathways and play a role in controlling calcium and phosphate homeostasis. Bone brittleness is caused by osteocyte cell growth failure, leading to osteoporosis [40,41].

Osteoclasts are multinucleated, large cells that are distinct from the hematopoietic lineage. They produce enzymes and acids that solubilize and digest minerals in the bones, and this process is referred to as resorption. Osteoclasts aid in the remodeling of damaged bones and developing pathways for blood vessels and nerves to pass through. Osteoporosis (increased osteoclast activity) and osteopetrosis (increased osteoclastic activity) are diseases marked by irregularities in osteoclastic activity [42,43].

Scaffolds, unlike permanent implants, are designed to offer temporary support for cell adherence. Scaffolds are able to replicate the complicated fibrillar design of natural ECM constituents and transport bioactive chemicals that stimulate tissue regeneration. Biomimetic scaffolds create a synthetic osteogenic milieu that aids ossification and improves therapeutic outcomes.

## 2. Nanofibrous Scaffold

A scaffold is an important element in tissue engineering for bone regeneration because it functions as a prototype for cell interactions and the development of bone extracellular matrix to offer structural support to newly produced tissue. Scaffolds are generally made up of a strong support structure coupled with a pore network. It must allow cells to colonize, proliferate, differentiate, and migrate. It should also have the requisite physicochemical qualities (such as strength, stiffness, biodegradability, and surface chemistry) for tissue development and the ability to tolerate and respond to mechanical stressors [44].

Natural bone has a one-of-a-kind mix of mechanical qualities due to an architectural design that spans nanoscale to macroscopic dimensions, with precisely and meticulously designed interfaces. Bone’s lightweight strength is due to the composite structure of mineralized collagen nanofibrils. The mineralized collagen fibrils subsequently align and organize in various ways to generate higher order structures and, finally, a complete bone [45]. With the capacity to create nanofibrous structures, a race has begun to replicate the ECM and create scaffolds that are an artificial extracellular matrix suited for tissue creation. Nanosized scaffolds were naturally created to resemble these nanofibrous collagen (a natural ECM). Nanofibrous scaffolds have fiber sizes that are similar to collagen fiber bundles, ranging between 50 and 500 nanometers [46]. By combining entirely regulated complex interconnected pore patterns and striving to emulate the 3D structure of natural collagen matrix, nanofibrous scaffolds address challenges of mass transfer (signaling molecules, nutrients, and metabolic waste movement) and spatial cellular organization (cell–cell and cell–matrix interactions). Furthermore, by employing a synthetic fibrillar matrix, these nanofibrous scaffolds eliminate the risk of immunogenicity and disease transmission [47]. However, surface, mechanical compatibility, and osteocompatibility are issues that need to be addressed.

**Figure 4:**
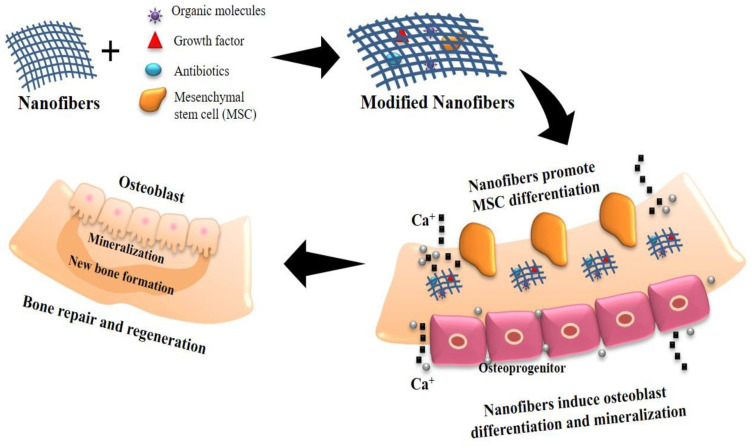
Mechanism of bone repair and regeneration induced by modified nanofibrous scaffolds.

Nanofibrous scaffolds have played a key role in biomedical research covering drug delivery to tissue regeneration because of their large surface area and the ability to control their characteristics by modifying formulation and fabrication parameters [48,49]. Nanofibrous scaffolds have different surface properties than standard or microstructured materials because of their substantially increased surface area and roughness and their dimensional resemblance to bone/cartilage tissue (such as wettability, surface energy, surface topography, and chemistry) [50,51]. The literature revealed that nanostructure scaffolds with cell-friendly surface properties facilitate more complex protein interaction. Due to their favored affinity for cell binding, apatite mineralization and osteoblast differentiation were promoted to stimulate new bone growth more than traditional materials (Figure 4) [52,53]. This may be one of the reasons why nanosized scaffolds outperform conventional materials in terms of tissue growth.

According to K.M. Woo et al., in an in vitro experiment, nanofibrous poly l-lactic acid (PLLA) scaffolds mimicked type I collagen fiber in size and outperformed solid-wall scaffolds in endorsing osteoblast proliferation and bone formation [54]. The researchers then delved further into the in vivo model to show that nanofibers can enhance the osteogenic potential and compared their effectiveness to solid-wall scaffolds in supporting bone regeneration. The scaffolds were inserted in critical-size rat calvarial bone defects. Nanofibrous scaffolds promoted significantly more new bone tissue development than solid-wall scaffolds, as evidenced by Von Kossa staining and micro-computed tomography measurements. Collagen deposition in nanofibrous scaffolds was confirmed by Goldner’s trichrome staining, but not in solid-wall scaffolds. Bone sialoprotein (BSP) and Runx2 were highly immunostained in the cells in these scaffolds. Trichrome, Runx2, and BSP were only weakly stained in solid-wall scaffolds implanted in the defects. Nanofibrous architecture improves osteoblast proliferation and bone development in vivo, according to these findings [55].

Various nanophase ceramic, polymer, metal, and composite scaffolds have been engineered by improving surface properties for BTR. The biopolymers that are broadly used in the electrospun nanofibers for bone tissue culturing and restoration include poly (hydroxyl acid) and poly (hydroxyalkanoates), such as poly (hydroxybutyrate) (PHB) and poly (lactic acid) (PLA). Gelatin, collagen, chitosan, and silk are natural polymers that can help in BTR [56,57].

## 3. Composition and Structure of Nanofibrous Scaffold

Scaffold architecture (such as porosity) can deliver adequate microenvironments for vascularization, cell migration, differentiation, proliferation, angiogenesis, and nutrient/waste exchange during bone regeneration. The hierarchical design of bone tissue is made up of nano-blocks (such as HA nanocrystals and type I collagen nanofibers). According to a recent study, different dimensional levels of nanostructures may have diverse functions in bone regeneration regulation. Nanofibers (2D level) can influence stem cell differentiation by altering cellular mechanotransduction mode and/or intracellular signaling in an indirect manner. Nano-featured surfaces have a larger surface area, which increases both protein and cell adhesion in bone regeneration. Increased protein absorption may result in more interfacial contacts among cells and substrates, most likely via integrin-mediated signaling pathways that influence cell activity (such as cell attachment, spreading, development, and osteogeneous differentiation) [58]. Porosity, pore size, and shape are also important factors in tissue engineering. Cell colonization and vascularization must be possible through pores. To ensure cell growth, the pore size should be kept between 200 and 350 µm.

**Figure 5:**
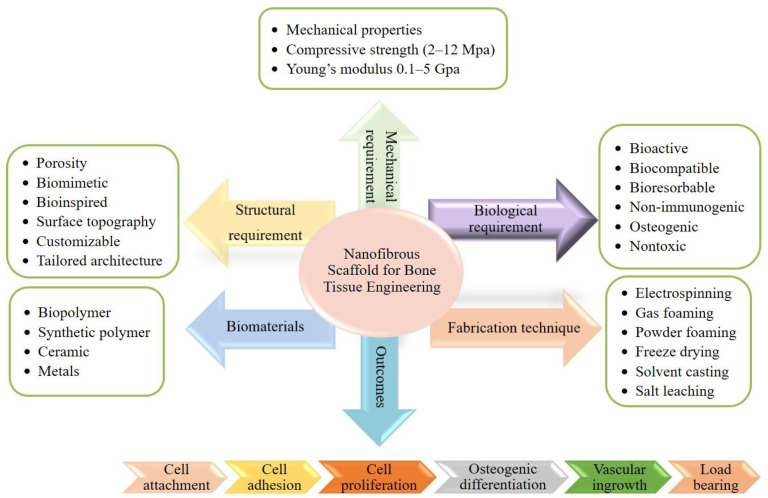
Properties, compositions, synthesis techniques, and outcomes of electrospun nanofiber for bone tissue engineering.

The nanofibrous scaffold is a three-dimensional (porous structures) cell matrix that acts as a basis for tissue regeneration. Scaffolds with pores of 200–300 µm showed the highest osteogenesis, perhaps since such pore size encouraged stem cell spreading and elongation [59]. Scaffolds having biocompatibility, durability, and biodegradability including chemically inert, high surface functionality possess adequate mechanical and physical properties that are ideal for tissue regeneration use. These scaffolds also facilitate cellular interactions and tissue growth, and represent a biomimetic template for managing new tissue growth (Figure 5) [60,61].

Scaffolds with hierarchical topologies spanning from nanometer to millimeter size have been designed to replicate the configuration and microstructure of the ECM. Metallic, hydrogel, or electrospun nano/microfiber scaffolds can be used to support bone tissue [62,63,64]. BTE research has sparked progress with new materials, manufacturing methods, performance assessments, and applications over the past few decades. Scaffold materials for structural support with optimal angiogenesis and osteogenesis properties have made significant progress [65]. Because of advances in scaffold fabrication and advanced technologies, bioresorbable scaffolds with regulated porosity and customized characteristics are now achievable. Using hydrophilic polymers to fabricate electrospun nanofiber scaffold is successful in developing fast-dissolving delivery systems with fewer drug–drug interactions. More calibrated nanomaterials have been researched in bone regeneration because these nano-structured scaffolds may provide numerous novel roles for the regulated discharge of growth factors or else cytokines to stimulate and control the surrounding cells for new growth of bone [66,67]. For the preparation of bone scaffolds, an extensive range of biomaterials has been investigated. Polymers obtained from natural or synthetic materials and hybrid materials are among them, with the selection criteria based on the scaffold’s desired physicochemical properties and the requisite biological cues [68,69].

Over the years, nanotechnology to enhance existing tissue and organ regeneration methods has achieved considerable attention. Nanomaterials can be used to build a fine structure (such as nanofibrous scaffolds) for tissue restoration, which is presently changing tissue engineering approaches in the medicine field [70,71]. Since the ECM assembly of bone comprises nanosized topographies, nanomaterials have been investigated to replicate the natural ECM structure of bone and facilitate effective regulation of cell behavior. As compared to conventional materials, nanomaterials have been found to advance osteoblast cell-adhesion and proliferation [72,73]. Nanofibrous scaffolds have drawn a lot of attention in BTR because of their high surface-to-volume ratio and the numerous ways to monitor their properties and applications. Nanofibrous scaffolds, in comparison to conventional microfibrous membranes and scaffolds, could reduce the inflammatory reaction when inserted into the defective area, speeding up the healing and regeneration of damaged tissue [74,75]. Progress has been made in the application of bioactive nano-ranged scaffolds for bone tissue repair and regeneration to improve the ability of scaffolds to simulate the complex features of the natural bone environment and provide a more conducive environment for cellular adhesion, growth, and new bone formation.

## 4. Manufacturing Process of Nanofibrous Scaffolds

Numerous techniques have been involved in the manufacturing of nanofibrous scaffold such as phase separation, self-assembly, electrospinning, and melt blowing. However, electrospinning is generally employed to develop nanofibrous scaffolds for biomedical applications. The nanofibers manufactured by the electrospinning method have a diameter ranging from 50 to 1000 nanometers. It is a simple, effective, and convenient technique for creating ultrathin smooth fibers [76,77]. The application of electrospun nanofibrous scaffold in BTE is the subject of this research and its technicalities are discussed in detail in the following Section 4.1.

### 4.1. Electrospinning: Method of Nanofiber Scaffold Fabrication

**Figure 6:**
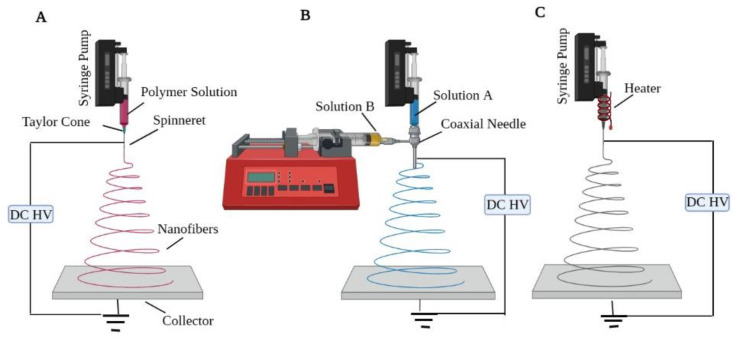
Schematic image of different electrospinning setups: (**A**) traditional electrospinning, (**B**) co-axial electrospinning, (**C**) melt electrospinning.

Electrospinning is a fast and efficient way to make nanofibers from natural and man-made polymers such as gelatin, cellulose, collagen, PVA, etc. In the 1930s, Anton Formhals laid the groundwork for the electrospinning process, which revived as a popular issue in the 1990s. Several research groups proved in the early 1990s that several organic macromolecules could be electrospun in the form of nanofibers [78]. A high voltage is used in a traditional electrospinning process to generate an electro-charge jet of molten/solution of polymer, which hardens on extrusion and forms a polymeric fiber. The three essential components of electrospinning setup/machine are a source of high-voltage power, a spinneret attached to a syringe/capillary tube (to deliver polymer solution), and a collector (Figure 6A) [79,80]. In a typical electrospinning arrangement, the spinneret is connected to a syringe containing polymer solution. Using a syringe pump, the solution can be charged at a consistent pace through the spinneret. An electric field is created among a counter electrode and a positively charged spinneret filled with a polymer solution in a conventional electrospinning setup. When a high voltage is supplied, the suspended drop of polymer solution at the spinneret’s nozzle is dynamically charged and the stimulated charges are uniformly spread throughout the whole surface. At equilibrium, the droplet’s surface tension would normally evolve into a spherical. The concentration of charge may cause a projection to form on the droplet’s end, reshaping it into a cone termed the Taylor cone. With a further rise in field strength, the electrostatic repulsion outweighs the surface tension, ejecting a charged jet of polymeric solution from the Taylor cone point once a critical level is reached. The polymeric solution is discharged as a jet, which then goes through a lengthening and twisting process, resulting in a long thin thread, which is finally gathered on a stationary or spinning grounded metallic collector. Two main electrospinning parameters greatly influence the fiber creation and structure: system parameters and processing parameters [81].

(a) System parameters include the physio-chemical characteristics of polymer solution such as conductivity, viscosity, surface tension, polymer molecular weight, and its disparity, which aid in the decrease in bead formation.
(b) Processing parameters include applied voltage, needle tip diameter, the flow rate of the pump, and needle-to-collector distance.

In addition to solution and processing parameters, other experimental configurations of the electrospinning process have been employed to vary the fundamental attributes of the fibers, such as collector type, use of multi-axial spinnerets, or conventional solution versus melt electrospinning. The collector type employed is a significant factor that may influence the architecture of the collected fibers. A basic collector plate will create an unraveling mat structure of fibers in a different alignment, while some collectors can gather oriented fibers. The rotational speed of generalized collectors such as spinning discs, drums, and mandrels is a crucial component that can impact the rate of fiber deposition. When a fiber connects to a collector and is pulled out of the collection, the fibers will extend or break into little segments if the collector moves faster than the fibers [82]. Co-axial or multi-axial electrospinning uses additional spinnerets for producing core-shell-type nanofibers compared to conventional techniques (Figure 6B). The co-axial arrangement consists of two spinnerets: an inner-core spinneret that is symmetrically wrapped around the outermost shell of a spinneret. After two polymeric solutions are concurrently introduced, a core-shell-type droplet is formed at the output of the interior and exterior nozzles. Core-shell nanofibers have been created with remarkable accuracy by using suitable solution concentrations. Polymers [83], biomolecules [84], proteins [85], and inorganic compounds [86] may all be restrained into the core element of core-shell-type nanofibers through co-axial electrospinning. Expanding the number of spinnerets aids in the formation of the core-shell structure, permitting the creation of the drug carriers for BTR. However, in spite of the ideal mechanical qualities and multifaceted architectures of electrospun nanofibers, the co-axial electrospinning approach provides inadequate control [87,88]. The melt electrospinning method improves on the traditional electrospinning approach by including a heat supply mechanism to generate the fibers (Figure 6C).

**Figure 7:**
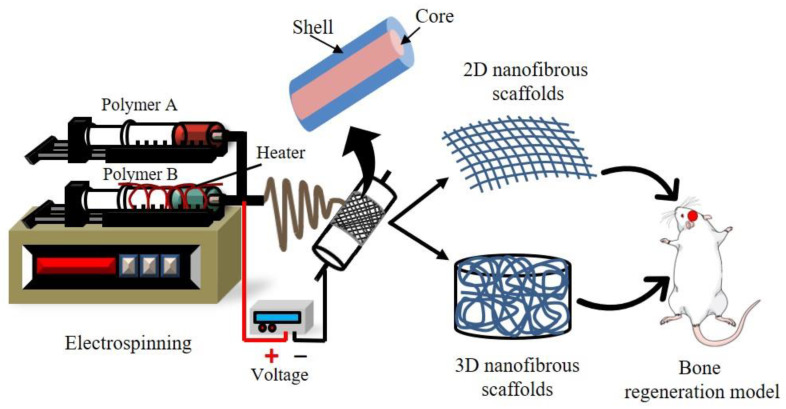
Diagrammatic representation of the production of 2D/3D nanofibrous scaffolds for bone regeneration using the melt electrospinning process.

Melt electrospinning is identical to standard electrospinning in function; however, the fiber conversion changes owing to the heat treatment. Rather than the solvent evaporation from the solution as in traditional electrospinning, the polymer is melted by the heating system, which subsequently cools and hardens [89]. Melt electrospinning has the potency to be exploited in BTE when solvent extraction and toxicity are challenges. Furthermore, because the fibers contain no leftover hazardous solvent, cytotoxicity is decreased in the melt electrospinning procedure. The ability to create three-dimensional nanofibrous scaffolds is one significant benefit that proves melt electrospinning a good choice for BTE (Figure 7) [90]. Owing to the wide range of fiber diameters (250–500 nm) that melt electrospinning can produce, it is feasible to construct three-dimensional structures that traditional techniques cannot. As a result, melt electrospinning may produce large fiber hole diameters, which has several benefits, including excellent cell invasion and development, as well as vascularization for innervated tissue such as bone. This technology has environmental benefits as well as increased production; nevertheless, the excessive temperatures necessary for melt electrospinning may not be feasible [91,92].

## 5. Natural Polymer Nanofibrous Scaffold

### 5.1. Gelatin

Gelatin (Gel) is a polynucleotide-based biopolymer obtained from animals, bones, tissues, and ligaments. It is regarded as an excellent biomaterial because of its low cost, biodegradability, and biocompatibility. RGD (arginine–glycine–aspartic acid) is the peptide sequence present in gelatin and it helps in cell adherence, growth, and migration of BMSC (bone marrow stromal cells) [93]. Gel’s properties have enticed several researchers to use it as a bone regeneration material; however, Gel decomposes quickly and has a low mechanical strength. Crosslinking, which can be conducted physically or chemically, can improve its mechanical properties [94]. In comparison to non-crosslinked scaffolds, electrospun Gel scaffolds that are crosslinked by genipin featured a diameter of 570 ± 140 nm and a stronger fiber structure, with confined fused patches where fibers intersected [95]. Gel as a natural biopolymer was eventually used to create a variety of drug delivery methods, including microparticles, nanoparticles, nanofibers, and hydrogels [96,97]. Nanoparticles of Gel are more effective for drug delivery to bone disease. Gel has versatile qualities as a drug delivery transporter because of its water absorbent and water-soluble properties [98]. Many bioactive compounds, such as HA nanoparticles, have been encapsulated in Gel to enhance osteoconductivity. BSA (bovine serum albumin) has been used as an exemplary protein drug, with a Gel concentration of 0.1% to 0.4%, resulting in a Gel solution with tremendous promise in BTE [99]. Gel-based electrospun nanofibers were shown to effectively initiate osseointegration and fast tissue development, in critical-sized bone damages. Xu et al. demonstrated a nanofibrous scaffold of Gel/β-TCP, wherein Ca^2+^ ions produced from β-TCP can cling to the carboxyl units of the Gel molecular chain, resulting in ionic-type interactions which stimulate osteoblast growth. Electrospun nanofibrous Gel/β-TCP has been demonstrated to have excellent biocompatibility and aid in the repairing of bone defects [100]. Salifu and colleagues examined the consequence of human embryonic osteoblast cells on crosslinked electrospun Gel/HA-aligned fiber scaffolds with varying HA contents [101]. Because of its qualities as a natural biomaterial and history of safe usage, Gel has been extensively researched as a drug delivery transporter across numerous drug groups in a varied range of medical and therapeutic uses [102].

### 5.2. Silk Fibroin

SF (silk fibroin) is a bio-macromolecule of protein complex [103,104]. It has been utilized as a biomaterial in the form of thin films, 3D scaffolds, electrospun fibers, hydrogels, and spheres in several biomedical applications, including BTE. SF-based nanoparticles are appealing in the study of drug delivery owing to their biocompatibility, nontoxicity, flexibility, elasticity, improvement of cell attachment and growth, chemical modification role, microbial resistance, low inflammatory response, and crosslinking capability [105]. Because of these characteristics, SF is the most reliable material in BTR. Membranes, microspheres, hydrogels, porous scaffolds, and nanofibers can all be fabricated from SF [106]. In the area of tissue defect rejuvenation, SF electrospun scaffolds are extensively researched for bone, brain, subcutaneous, etc. SF, as a biomaterial for BTR, not only induces ECM and is compatible with cells, but it may also stimulate the formation of HA crystals, which leads to the integration of bone. SF, being an osteogenic biomaterial, has the capacity to promote stem cell development by blocking the Notch pathway [107]. Kirker-Head and co-workers fabricated silk scaffolds that have been proven to be an osteoconductive mold for repairing critical size mid-femoral segment deformities in nude rats [108]. The use of SF to deliver BMP-2 to be used in bone regeneration has been extensively researched. In vivo, SF mixed with BMP-2 growth factors and HMSCs (human mesenchymal stem cells) improved osteoblastic adhesion and differentiation, increased ALP staining, and encouraged bone growth [109]. SF is typically used in concert with other biomaterials that have been shown to assist BTR, such as inorganic components consisting of calcium phosphate or collagen, both of which are naturally present in bone [110]. In a rabbit model, the fusion of HA nanoparticles into silk matrix improved bone repair [111]. In vitro bone regeneration was achieved using electrospun SF/PLCL nanofibrous scaffolds cultured with hADSCs. The tensile strength of the SF/PLCL (50/50) scaffold was (6 MPa). Furthermore, the aptitude of Silk fibroin to indorse osteogen differentiation of the hADSCs (human adipose-derived mesenchymal stem cells) was proven by its elevated ALP activity, which had an absorbance index with value of 150 when matched to pure PLCL (with an absorbance index of 80) [95].

### 5.3. Collagen

Collagen (Col) is a biocompatible and bioactive polypeptide molecule that represents about 25% to 35% of the complete body protein and has a characteristic molecular structure and fibrillar structure. It helps support extracellular scaffolding, which represents a significant component of ECM in numerous connective tissues, including bone [112,113]. Col is a category of naturally occurring proteins that make up the majority of connective tissue. Col-I is the utmost prevalent kind of collagen in the human body. Col-I is biodegradable, antigenic, and has beneficial properties such as angiogenesis stimulation and prevention, along with improved cellular proliferation as well as differentiation promotion. Due to these qualities, Kumar et al. proposed that Col has become a new preferred substrate material for a variety of bio-degradable tissue engineering and regenerative medicine applications. These researchers created a multilayer nanocomposite of nHA-Col to examine the influence of Col. The results showed that scaffold specimens improved seeded MSC adhesion, growth, and differentiation [114,115]. Col scaffold enhanced cell growth in vivo [116]. Because of its safety and biocompatibility, Col-based drug delivery scaffolds are commonly utilized as templates to stimulate bone regeneration. Col-derived scaffolds created through electrospinning have a 3D microstructure that can be employed to induce tissue regeneration effectively. Fischer et al. created Col/HA scaffolds that may be exploited in tissue engineering scaffolds to stimulate cell development and enhance cellular adhesion [117]. A permeable Col-based scaffold was treated with Sulfo-SMCC (Sulfosuccinimidyl-4-(*N*-maleimidomethyl) cyclohexane-1-carboxylate) and Traut’s reagent to increase BMP-2 adhesion to Col scaffolds. BMP-2 release slowed by crosslinking, but its biological activity was not affected. In the in vivo investigation, the use of Sulfo-SMCC and Traut’s reagent to chemically link Col scaffolds with BMP-2 was found to be an excellent delivery approach for bone development and BTE [118]. To assess osteointegration, cell adhesion, propagation, and differentiation were assessed in Col-I/PLLA and HA-modified scaffolds. The results reveal that a segmental bone defect may be repaired 8 weeks after surgery utilizing nHA/Col/PLLA reinforced with chitin fibers and seeded with cultured goat bone marrow MSCs [119].

### 5.4. Chitosan

Chitosan (CS) is a polysaccharide class of biopolymers that consists of a β-(1-4)-linked 2-amine-2-deoxy-β-d-glucose monomeric parts. CS is normally gained by deacetylation of the chitin that is derivative of fungus cell walls, arthropod and insect exoskeletons, mollusk radulae, and cephalopod beaks. Because of its admirable traits such as high charge density and being biocompatible, biodegradable, non-toxic, antimicrobial, non-carcinogenic, and simple to make, it was investigated as a factor in drug delivery applications in tissue engineering and pharmaceutics [120,121]. Some studies have shown that CS improves cell adhesion, proliferation, osteoblast differentiation, and mineralization. This activity is linked to the scaffold’s physical properties as well as electrostatic interactions (caused by CS’s cationic nature) with a variety of chemicals, including cytokines and GFs. These substances help cells colonize more effectively [122,123].

However, pure CS has weak mechanical properties, lacks osteogenic inductivity, and lacks natural bone properties. To overcome this limitation, CS scaffolds can be combined with other natural or artificial macromolecules (alginate, Gel, Col, SF, PVA, PCL, PVP, etc.) and biomaterials (β-tricalcium phosphate, SiO_2_, HA, etc.). Calcium phosphate particles and HA nanoparticles could be mixed with CS matrix to create CS-based composites that imitate real bone. The qualities predicted for CS/calcium phosphate scaffolds include biocompatible, biodegradability, osteoconductive, antimicrobial properties, osteoinduction, angiogenesis control, and mechanical strength. Several investigations using CS/HA composite materials for BTR have been accomplished [124,125]. Zhang et al. found that when nHA/CS composite scaffolds are placed into the segmental bone lesion, the bone regeneration rate is greater than pure CS scaffolds. For the investigation, critical-sized bone defects (length: 10 mm, diameter: 6 mm) were fashioned in the left femoral condyles of 43 healthy New Zealand white rabbits. The femoral condyle deficiencies were treated with nHA/CS scaffold implantation, pure CS implantation, or left unfilled. The data show that 12 weeks following the treatment, full repair of the segmental bone defect was seen in rabbits implanted with the nHA/CS scaffold, whereas the defect remained visible in the CS-only group [126]. Composite of HA/CS may also be employed as a functional coating on further implants to develop biomaterials with outstanding osteoinduction capabilities. Wang et al. investigated that a coating of HA/CS on a titanium surface is a favorable approach for producing biomaterials with improved osteointegration potential and tested it in diabetic patients. The histological analysis at the bone–implant interface demonstrated that after four weeks, tiny regenerating bone was incorporated into Ti/cTi. After twelve weeks, greater bone contact was detected, plus a greater volume of new bone formed in the cTi implant compared to the Ti implants. CS could be layered on top of metal (Ti) implants to increase osteointegration [127]. Sharifi et al. created a CS/PCL composite scaffold and then conducted an MTT experiment using human osteoblast (MG-63) cells to assess its cell proliferation. The result demonstrated that scaffolds are biocompatible, promoting proliferation, and can be an outstanding contender for BTE application [128]. Chen et al. used coaxial electrospinning to create Gel–CS/HA core-shell nanofibrous scaffolds. Inside the core-shell nanofibrous scaffold, CS and Gel promote cell attachment and proliferation, which is aided even more with the existence of HA deposition on the surfaces of nanofiber. When equated to CS, Gel, and CS–Gel composite nanofibers, core-shell CS–Gel nanofiber scaffold boosted HA mineralization efficacy and designed a homogeneous HA deposit. The results of an MTT experiment using human osteoblast (MG-63) cells cultivated on core-shell nanofibers reveal that HA deposition on the core-shell CS–Gel nanofibers may also promote osteoblast cell growth [129]. CS and alginate can be mixed to form a polyelectrolytic complex that results in mutual precipitation and increased mechanical strength. CS provides structure to the supports, whereas alginate aids cell regeneration [130].

## 6. Synthetic Polymer Nanofibrous Scaffolds

### 6.1. Polycaprolactone

PCL (Polycaprolactone) is a biodegradable, biocompatible, and bioabsorbable linear aliphatic poly-ester [131]. It could be exploited to make scaffolds for applications in tissue engineering such as bone or cartilage regeneration, surgical sutures, and drug delivery systems, among others [132,133]. PCL, being a semi-crystalline polymer, has a melting point between 55 °C and 60 °C and a glass transition temperature of −54 °C. It maintains a rubber-like state of good material penetrability under physiological parameters [134]. PCL nanofiber is a potential option for BTR and drug delivery because of its comparatively low degradation rate and high modulus value. Microorganisms and hydrolytic, enzymatic, or intracellular mechanisms all can degrade PCL under physiological conditions; however, when compared to PLA, PGA, and PLGA, PCL shows a sluggish degradation rate of 2 to 4 years. Because of its hydrophobic nature, it is more appropriate for long-term implants and drug delivery than general tissue regeneration. According to the latest report, gravity-spun nanofibers of Col-coated PCL increased human osteoblast cell proliferation rates [135]. PCL has also been effectively employed to entrap antibiotic drugs, and it has been developed as a drug delivery mechanism for promoting bone growth and redevelopment in the healing of bone deformities [136]. Yao et al. created 3D nanofibrous scaffolds of PCL/PLA, having the mass ratio of 4:1, with great porosity (96%). In in vitro tests, PCL/PLA scaffolds enhanced human MSC’s osteogenic differentiation, cell proliferation, apatite-like deposition, and osteogenic gene expression and demonstrated homogeneous scaffold cellularity. In vivo experiments presented findings of fresh bone development in mice of critical size; when the same amount of rhBMP2 (0.75 gm) was applied to each scaffold group, the PCL/PLA-rhBMP2 group (4.56%) had more bone formation than the PCL-rhBMP2 group (0.99%) [137]. Rezk et al. fabricated Mg (magnesium)-coated PCL/HAp/SIM nanofiber. In vitro cell culture test showed that incorporating HA nanoparticles and SIM (simvastatin) into the composite nanofiber improved osteoblast cell adhesion and proliferation, indicating that the nanofiber has great capability for bone cell regeneration [138]. Altogether, PCL is a biodegradable biopolymer that deserves further research as a permeable scaffold for BTR and drug delivery uses.

### 6.2. Poly (Lactic-co-Glycolic Acid)

PLGA (poly (lactic-co-glycolic acid)) is one of the most attractive biocompatible and biodegradable synthetic copolymers of poly-lactic acid (PLA) and poly-glycolic acid (PGA), more effective in the fabrication of nanofibrous scaffolds for many biomedical applications [139]. When the polymers contain a 50:50 ratio of glycolic and lactic acid monomers, the breakdown rate is fastest and one of the most commonly utilized polymers in nanomedicine [140]. PLGA has biocompatibility and biodegradability properties, making it a good drug delivery carrier and protecting drugs from degradation [141]. In biomedical applications, PLGA particles have the advantage of protecting DNA and other biomolecules from decomposition and improving immunological response effectively [142]. In vitro culture of the MG-63 osteosarcoma cell line revealed that PLGA/zeolite 7% *w*/*v* scaffolds provided favorable conditions for cell proliferation and activity. PLGA/zeolite nanofibrous scaffolds provide a novel approach to regenerate bone tissue with good biodegradability and cell compatibility [143]. Another study shows that incorporating nHA into the PLGA microspheric scaffolds could advance the bioactivity of the scaffold designed for BTE. In vitro studies have proven that PLGA/nHA composite nanofibrous scaffolds improve rabbit MSC proliferation, differentiation, and mineralization [144].

### 6.3. Poly (Lactic Acid)

Another biodegradable material that has been widely employed as an intricate material for tissue engineering scaffolds is PLA, poly (lactic acid). It is a linear type of aliphatic thermoplastic polyester made by ring-opening polymerization (ROP) of the lactide with direct poly-condensation of the lactic acid and is found in three isomeric forms: D-PLA, L-PLA, and a racemic combination of D- and L-PLA [99,145]. PLA’s melting temperature, Tm, is between 170 °C and 180 °C, with a glass transition temperature, Tg, of 50–65 °C [146]. PLA can be mixed with various polymers to improve associated qualities or create new PLA polymers/blends to modify tissue-specific scaffolds [147]. PLA nanofiber scaffolds have been broadly used in the bio-medical area, primarily in applications such as drug delivery vehicles, bone fixation material, and sutures, due to their biocompatibility, biodegradability, and acceptable mechanical properties. The surface characteristics of dexamethasone-loaded multilayer nanofibrous PLA composite scaffolds have been discovered to be appropriate for BTE and medication delivery. The best osteogenic propagation and differentiation capacity were found in electrospun multilayer scaffolds with medicines in the central layer [148]. Three-dimensional (3D) electrospun fibrous scaffolds have indeed been offered as feasible tissue engineering approaches. Ye and colleagues created 3D macro-porous nanofiber scaffolds for BTE. Pre-fabricated electrospun nanofibers with a mix of freeze-drying, homogenizing, and thermal crosslinking procedures were used to generate 3D nanofiber scaffolds made of nHA/PLA/Gel. Peptides derived from BMP-2 were then immobilized on three-dimensional scaffolds with the assistance of polydopamine (pDA) coating, resulting in 3D nanofiber nHA/PLA/Gel-PEP scaffolds; the release of BMP-2 peptides might be sustained. In an in vitro investigation, nHA/PLA/Gel-PEP scaffolds enhanced the effectiveness of BMSCs’ alkaline phosphatase and gene expression linked to cell proliferation and osteogenic differentiation. In the rat cranial model, the scaffold was tested in vivo, and radiography and histology studies revealed that it promoted bone formation in the defects [149].

### 6.4. Polyvinyl Pyrrolidone

PVP (polyvinyl pyrrolidone), often referred to as povidone or polyvidone, is a linear manmade polymer comprising monomers of 1-vinyl-2-pyrrolidone. The PVP backbone is made up of highly hydrophilic pyrrolidone units and relatively hydrophobic alkyl units [150]. It is a tasteless, hydrophilic, chemically inert, non-toxic, biodegradable, and non-ionic polymer. It comes in the form of slightly pale to yellowish waxy flakes of varying molecular size and weight. It has long been known for its amorphous nature, rapid dissolution in organic solvents, and propensity to interrelate with hydrophilic substances [151]. PVP is an effective biomaterial due to its inherent qualities such as coating ability, adhesiveness, pH stability, resistance to high temperatures, mechanical strength, crosslinking, and excellent complex formation capacity. PVP has long been used in the biomedical area as an excipient, dressing material, drug delivery tool, drug coating material, etc. PVP, one of the most frequently employed manmade polymers, is utilized to make nanofibers and nanoscaffolds for biological applications such as tissue engineering [152,153]. For bone tissue graft applications, Uma Maheshwari et al. developed pure HAp, TCP, and HAp and TCP embedded PVP/PVA blended nanofiber composite scaffolds. SEM, EDAX, XRD, DSC, and FTIR were used to characterize the synthesized scaffolds’ physical, chemical, and thermal characteristics. MG-63 cell lines were used to test and compare the biocompatibility of the produced scaffolds [154]. The core-shell-type nanofibers were created by the electrospinning a cellulose acetate–PVP composite (a core made of cellulose acetate and a shell of PVP). When the PVP concentration was increased from 0% to 2% weight, homogeneous and cylindrical nanofibers were generated. However, the flattened nanofibers were formed when the concentration was increased to 5% by weight. These HA-loaded composite nanofibers were discovered to be prospective materials for BTE [155]. 

## 7. Hybrid Nanofibrous Scaffolds

Polymeric scaffolds provide a suitable physical milieu for the regeneration of bone tissue. A tissue scaffold’s bio-stability may be elucidated by paying close attention to three key factors: strength, elasticity, and the absorption of biomolecules onto the material surface. When a scaffold is implanted, its mechanical qualities must be maintained, especially when it comes to major load-bearing components such as bones. Additionally, the blended scaffolds will improve mechanical characteristics, resilience against degradation, and affinity for biological components. Here, we discuss some natural/synthetic polymer blend nanofibrous scaffolds frequently used in BTE.

Shalumon et al. designed CS/PCL hybrid nanofibrous scaffold, which showed significant cell attachment and proliferation. The study found that combining CS with PCL improves the scaffold’s hydrophilicity and its potential for degradation [156]. It was discovered that the elasticity and stiffness of the scaffold were caused by PCL and depolymerized CS, respectively, when PCL/depolymerized CS was electrospun at various ratios. This combination was better since it did not employ crosslinkers, which might eventually have cytotoxic consequences [157]. Yang et al. also assessed electrospun PCL/CS nanofiber and showed that adding CS into PCL can improve Young’s modulus, fiber diameter, and hydrophilicity. In 2D and 3D cultures, osteogenic differentiation of preosteoblasts was tested using PCL nanofibers containing varying levels of CS (0%, 3%, and 9%). Compared to PCL placebo nanofibers, the addition of CS enhanced calcium deposition, ALP activity, and the expression of osteopontin (OPN), as well as the adhesion and proliferation of MC3T3-E1 cells [158]. One of the findings showed that blending PCL with Gel altered the distribution of fiber diameter and pore size. Blended Gel enhances the mechanical and morphological characteristics of the fibrous network and has a positive impact on cellular response. Moreover, the findings imply that the PCL/Gel blend improves the polymeric scaffold’s cellular and mechanical properties. Overall, these results show that blended PCL/Gel scaffolds are better than their PCL counterparts [159]. For example, Ren et al. [160] fabricated a PCL/Gel hybrid nanofibrous scaffold that exhibited good tensile strength and biocompatibility in MC3T3-E1 cells and enhanced the osteogenic capability. The mechanical characteristics of PDLA (Poly-d-Lactide)/PLLA/Gel electrospun fibers have been studied and the results demonstrated significantly increased mineralization of the bone tissue as an increase in the mechanical properties of the fiber [161]. A PLLA/Gel blend nanofiber scaffold significantly mirrored the bone’s ECM by changing its physical and biochemical characteristics [162]. In addition, biopolymer blends of PVA and SF have been fabricated as nanofibrous scaffolds by the electrospinning technique and showed potential for BTE. Kobori et al. [163] investigated the properties of SF/PVA blend fibrous scaffolds and found that the proliferation of MG-63 cells on scaffolds was significantly high, and greater tensile strength was observed with an increase in SF concentration.

In this context, Table 2 shows the findings of numerous current investigations on polymer usage in BTE conducted over the past ten years.

## 8. Nanofibrous Composite Scaffold

**Figure 8:**
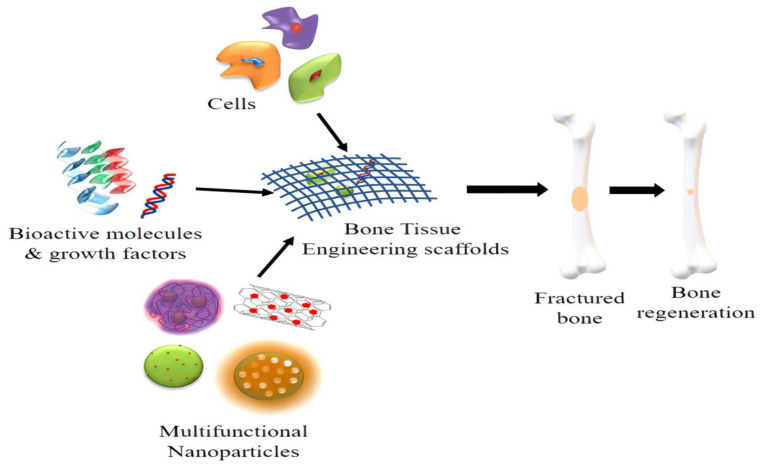
Nanofibrous composite scaffolds for bone tissue regeneration.

Tissue engineering, enzyme immobilization, and drug/biomolecule delivery could all benefit from functional nanofibrous scaffolds created by electrospinning. Using a mixture of multi-component formulations and electrospun scaffolds, the bio-chemical and physical qualities of nanofiber scaffolds should be modified to fit the context for a particular successful application [177,178]. Bone scaffolds should encourage angiogenesis (the development of new blood vessels) and be biocompatible to ensure appropriate blood circulation at the implant site. Scaffold biodegradation or bioresorption should be executed in a regulated manner over time, so that it not only provides a surface for the development of new bone and vasculature during regeneration, but is also replaced once the bone defect is entirely repaired. Several organic and/or inorganic particle mixtures were investigated as composite nanofibrous scaffolds that improve the advantages and lessen the downsides of each constituent, which can efficiently improve tissue regeneration (Figure 8) [179,180].

Many researchers have built plans of different composite scaffold schemes having diverse materials to address the drawbacks of a single particle. The production and trends of functional scaffolding biomaterials, such as nanocomposites of HA, carbon nanotubes (CNTs), and magnetic nanoparticles with various polymers, are summarized in this section.

### 8.1. Ceramic Nanofiber Scaffold

Calcium silicate, bioactive glass (BG), tri-calcium phosphate (TCP), and HA are examples of ceramics/bioceramics [181,182]. Ceramic composites are the most recognized composite materials for BTR [183]. Ceramic nanoparticles are composed of carbides, oxides, carbonates, and phosphates of metals and could be used in the formation of nanosized materials of many shapes, sizes and porous structure [73,184]. Several studies have found that nanoceramics with smaller pore sizes stimulate osteochondral development, osteogenesis, and vascularization more effectively. The benefit of ceramics is that they are biocompatible with the human body. Due to numerous favorable properties, such as high chemical inertness and heat resistance, they are broadly used in the applications of BTR. Ceramic nanoparticles are known to be effective transporters for medicines, DNA, proteins, and imaging agents in the biomedical area due to their chemical/physical characteristics [120]. Many ceramic biomaterials composed of calcium phosphate and mineral trioxide combined materials have been used for BTR because of their excellent biocompatibility, biodegradability, and similarity to the inorganic components of bone minerals [16,185]. The most promising biomaterials are bioactive glass ceramic (BGC), which are extensively applied as a filler material for BTR. Bioactive glass nanoparticles (BGNs) are attractive candidates for orthopedic applications [15,186,187]. Bioactive glass nanoparticles could attach to the bone surface and change into a bone mineral-like apatite phase deposited on the surface, increasing bone graft bioactivity, osteogenic differentiation, and cellular mineralization.

Osteoblast adhesion and proliferation are aided by bioactive ceramics. On the other hand, bioceramics have had a limited effect on clinical success because of their fragile behavior, sluggish deterioration, and complexity in forming precise structures [133,188]. The bioabsorbable ceramic material TCP is well known and when TCP is implanted into the body, it has been shown to regenerate bone structure and bond with the bone. As a result, designing scaffolds containing bioceramics can be a suitable method for simulating bone natural composition. The intrinsic flaws of bioceramics can be resolved via a composite nanofiber scaffold [182,189]. Calcium phosphate nanoparticles embedded in Gel/PCL nanofiber scaffolds improved mechanical properties and accelerated apatite crystal nucleation and development [190].

HA is the most commonly utilized ceramic bone graft and a suitable coating layer for orthopedic implants. It is found in two forms, natural HA and synthetic HA [191]. Other combinations of biomaterials have shown that incorporating HA with scaffold creates the most favorable microenvironment, thoroughly mimicking the natural condition of bone [192]. Nie et al. and others, including Fu et al. [193,194], investigated the discharge pattern of BMP-2 (bone morphogenetic protein) via nanofiber scaffolds of PLGA/HA. Scaffold encapsulation efficiencies varied from 49% to 66%. The study revealed that enhancing the amount of HA nanoparticles in the scaffold accelerated the discharge summary of rhBMP-2 (recombinant human bone morphogenetic protein-2) and protected BMP-2 from deterioration. In vitro, the incorporation of HA can enhance cell attachment and viability. In vivo experiments revealed that PLGA nanofibrous scaffolds simply filled by BMP-2 did not affect bone defect. When nanoparticles of HA were hooked on the PLGA during the electrospinning process, BMP-2 was secreted from the scaffolds (here, BMP-2 was encapsulated into nanofibers or coated on nanofiber surface), which led to improved bone repair. These findings indicate that the existence of HA nanoparticles was critical for the sustained discharge of BMP-2 rather than GF’s initial loading position.

### 8.2. Carbon Nanotube Nanofiber Scaffold

Since their discovery in 1991, carbon nanotubes have been considered the most promising for biomedical uses. Many studies in recent years have shown that carbon nanotubes (CNTs) could be an appropriate scaffold material for BTE because of their high strength and low weight. They have also received attention for developing revolutionary methods for treating bone diseases such as myelomatosis, osteoporosis, non-union bone deformities, and bone cancer because of their unique features, such as CNT-based delivery systems [195]. Carbon-based nanomaterials such as CNTs have been introduced into various polymer matrices such as Col, CS, and PCL to improve mechanical strength and osteogenic differentiation capacity for BTE. CNTs are favorable to material exchange of ECM in bone tissue because of their interconnected nano-network structure and suitable porosity. Their adaptable surface chemistry and high affinity for cell-binding proteins can be utilized to control cell shape and enhance the differentiation of stem cells into osteocytes, particularly osteoblasts and neural lineage cells [195]. CNTs are an allotropic type of carbon that are hollow cylinders made entirely of graphitic carbon sheets that are rolled as single-walled (SWCNTs), doubled-walled (DWCNTs), or multi-walled (MWCNTs) structures [196,197,198]. CNT diameters must always be kept in the nanometer scale (0.5–2 nm for SWCNTs, 3 nm for DWCNTs, and 2–100 nm for MWCNTs), but their lengths can easily surpass microns. They are mechanically and chemically stable and have excellent electrical properties. Despite their appealing potential, CNTs have significant drawbacks in biomedical applications [199]. In combination with structural and mechanical limitations of substrates made entirely of CNTs, CNT toxicity remains a major issue restricting their biological use. Polymers have been carefully investigated as a more favorable and flexible medium for incorporating CNT in biological systems to help both aspects [200,201]. As a result of these findings, a diverse range of 3D architectures has been developed (i.e., scaffolds).

CNTs are being used in the manufacture of bone-like materials. The elastic modulus and compressive strength of 45S5 bioglass scaffolds are increased by MWCNTs, as well as the strength and tensile modulus of CS films and the surface irregularity of composite scaffolds of CNT-PLGA [202,203]. SWCNT/PLGA composites formed by incorporating SWCNT into a PLGA matrix also showed superior properties. When compared to PLGA-only scaffolds, SWCNT/PLGA composites have a higher MC3T3-E1 cell proliferation rate, as well as a higher compressive modulus and ultimate compressive power [204].

### 8.3. Magnetic Nanofiber Scaffold

Magnetic nanoparticles (MNPs) and magnetic fields are being used in novel ways to enhance bone repair performance, including targeting, cell labeling, designing, and gene modifications towards building scaffolds, growth factor carriages, and cells [205]. The encouragement of signaling streams having integrin, MAPK, NF-B, and BMP was recognized as part of the procedure of scaffolds comprising MNPs utilizing magnetic fields and stem cells to boost bone restoration [206,207].

Magnetic fields combined through signaling and GFs, magnetically assisted stem cell freezing and refreezing, and magnetically supported scaffold and coating assemblages can all help with bone regeneration. According to studies, static magnetic fields (SMFs) of moderate strength increased bone mineral compactness and bone repair in animals [208]. Iron, nickel, cobalt, and their oxides can be utilized as MNPs in biomedical procedures for drug delivery, 3D cell organization, diagnostics, cell monitoring, and biosensors. Some MNPs have superparamagnetic characters, allowing for enduring, non-invasive monitoring [209,210]. SPIONs (superparamagnetic iron oxide nanoparticles) have potential applications in cell monitoring, targeted drug administration, gene therapy, imaging, tissue engineering, and hyperthermia. SPIONs can increase tissue healing efficiency, be accountable for complex mechanical stimuli for bone rejuvenation, promote osteogenic differentiation of BMSCs, and establish bone remedial in vivo without a magnetic field [211]. However, MNPs are cytotoxic, and coating is needed to overcome their cytotoxicity and produce biocompatible MNPs due to safety concerns; hence, it is better to combine them with bio-friendly structures such as scaffolds.

A nanofibrous Fe_2_O_3_/HA/PLA scaffold was generated in an experiment. Because of the SPION integration, this scaffold enhanced osteoblast proliferation [180]. In another study, SPION/Gel scaffolds were inserted in the incisor openings of rats, which enhanced the bone repair over porous Gel scaffold control lacking SPIONs [212]. MNPs can use magnetic fields to transmit biological mediators such as chemotherapeutics, medicines, antibodies, oligonucleotides, peptides, and GFs.

## 9. Growth Factor-Dependent Nanofibrous Scaffold for Bone Tissue Regeneration

The universal aim for BTE exists in creating a scaffold that functions identically to natural ECM, which can be accomplished by adding factor GF or modifying nanofibers. The three chief modules of tissue engineering are stem cells, biomaterials, and GFs. GF delivery plays a vital function in tissue redevelopment and GFs are being used in new clinical policies to improve the healing of broken bones, limit disproportionate bone development, and hasten the curing process, and they usually advance the distribution of therapeutics [16]. These factors facilitate the migration of progenitors and inflammatory cells to commence the healing process in a bone regeneration environment by diffusing signals at the defect site through the ECM. As a result, adding GFs to the scaffold biomaterial is expected to promote osteogenesis and angiogenesis while also controlling excessive bone growth and speeding up the healing process. According to their structural and evolutionary properties, GFs can be divided into numerous families. Some of the GFs addressed in the present review for BTE purposes are BMP (bone morphogenetic protein), VEGF (vascular endothelial growth factor), TGF-β (transforming growth factor), PDGF (platelet-derived growth factor), and FGF (fibroblast growth factor). Each of these GFs has a distinct role to perform in bone repair. Co-axial electrospinning/emulsion electrospinning, layer-by-layer (LBL) multilayer assemblage, physical adsorption/encapsulation, enclosed in micro/nanosphere and chemical deposition via photo-immobilization, click chemistry, and plasma treatment are the five procedures for loading GFs on nanofiber scaffolds [112]. GFs are signaling polypeptides or proteins, which are released soluble. These GFs govern undistinguishable cell proliferation and differentiation by binding to their explicit transmembrane receptors and conveying intracellular signals to increase or decrease particular cell functions. GFs are commonly employed in the human body to activate endogenous proteins which stimulate cell proliferation and differentiation [213]. The TGF-β series includes GFs and BMPs, which impact cellular activity, growth, differentiation, and ECM production in a variety of cells. Twenty BMPs, including BMP-2/-4/-5/-6/-7, have been identified as being involved in osteogenic differentiation and efficiently endorse the differentiation of BMSCs hooked on osteoblasts and encourage bone formation; among them, BMP-2 and BMP-7 have attracted a lot of attention, and have been integrated in FDA-approved bone regeneration devices [16,214]. BMP-2 is a bone-promoting GF with a nanosize of 7 × 3.5 × 3 nm^3^. BMP signaling is believed to be involved in a large number of cellular functions, with canonical signaling transduction by BMPs occurring via both Smad-dependent and MAPK (mitogen-activated protein kinase) signaling pathways. BMP regulates osteogenic activity, which has been found to enhance transcription of osteogenic marker genes such as Runx2. Notwithstanding their excellent potency, recombinant BMPs have several issues with their clinical application due to their short life and rapid clearance by body fluids. Since proteins injected into the affected site lose their biological function over a certain amount of time, to accomplish adequate bone regeneration, large dosages of recombinant BMPs are employed in medical research. The large amount and multiple administrations of BMP-2 might cause undesirable systematic side effects. To avoid these issues, drug delivery systems must be developed to safely regulate their biological activity for a prolonged period, prevent destruction, and monitor their slow release to the intended location [215]. Kim et al. created PCL/Gel/BCP (biphasic calcium phosphate) scaffolds loaded with BMP-2, and the discharge of BMP-2 was shown to contribute to initial bone development [216].

PDGF, which is released by platelets at the area of fracture all through initial tissue regeneration, is a messenger protein that, like BMPs, plays a crucial role in bone healing. There are multiple isoforms comprising PDGF-AA, PDGF-BB, PDGF-AB, PDGF-CC, and PDGF-DD. Among them, PDGF-BB, which can attach to every isoform of the PDGF receptor, is regarded as the universal GF in this family. The inclusion of PDGF-BB into nanofibrous scaffolds was found to be beneficial in enhancing bone cell proliferation and mesenchymal cell migration concurrently in various studies [107]. In an in vitro study, Raghavendran and other co-authors found that PDGF-BB functions synergistically with biomaterials such as PLLA/Col/HAp as well as PLLA/HAp to increase the potential of osteogenic differentiation. As a result, this combination can be employed to regenerate bone tissue [217].

Other GFs are FGF-1 and FGF-2, which are the most reported GFs during regeneration of bone, which induces angiogenesis by enhancing callus development and osteoblast cells. It has been reported that specifically, FGF-2 signaling peptides implicated a strong angiogenetic action, increasing the number of osteoblasts and chondroblasts, resulting in bone regeneration [218,219,220]. Rubert M. et al. successfully fabricated PCL/PEO coaxial fibers containing FGF-2, which displayed long-term release, and established its capacity to increase fibroblast cell survival and proliferation [221].

VEGF is an essential cofactor of angiogenesis in bone growth, particularly VEGF levels peaking in the days after a bone fracture. It is a signal protein and is essential for the propagation, relocation, and instigation of the endothelial cells such as paracrine factors. Moreover, its bioactivity endorses angiogenesis, cell differentiation, and chemotactic activity. Because of its capacity to stimulate neovascularization (angiogenesis) and improving bone vascularity, VEGF is of particular interest. Furthermore, it is vital in the enhancement of blood vessel permeability and fenestration [60,222]. In mouse femur fractures, VEGF can improve blood vessel production, ossification, and new bone (callus) maturation, as well as increase bony bridging of a rabbit radius segmental gap defect. Rosa and co-workers demonstrated that scaffolds made of PLGA/BSA/VEGF increased cell adherence and were innocuous to cells [223]. Table 3 contains an overview of the literature reviewed in this section.

## 10. Conclusions and Future Prospects

Complex bone fractures produced by a variety of traumas and diseases require intervention. Autografts are still the “gold standard” in orthopedics due to their effectiveness and biocompatibility, but they are in short supply. Therefore, it is the need of the hour to develop alternatives for bone healing which are safe, cost effective, and show rapid bone repair. Tissue engineering is a new interdisciplinary research area to restore or improve tissue structure, and it can significantly raise the quality of human life. Scaffolds used in tissue repair should have a chemical composition and physical structure similar to native ECM. Blood vessels deliver nutrients, oxygen, and neurogenic GFs to stimulate neurogenesis, whereas nerve fibers deliver vascular neuropeptides in a common pathway to enhance angiogenesis, ultimately promoting bone development and repair in a synergistic manner. The osteogenesis and neurogenesis of hBMSCs are influenced by the brain-derived neurotrophic factor (BDNF). Increased neurogenesis may be a way for BDNF to indirectly enhance osteogenesis. This review chiefly engrosses the fundamentals of nanofibrous scaffolds for BTR to provide investigators with a universal understanding of their potential applications in BTE. Natural and semisynthetic polymers are popular because of their biodegradability and simplicity of production, such as PLGA, PGA, PLA, PLLA, CS, Gel, and Col, known as exceptional contenders for biomimetic scaffolds for BTR uses. Electrospinning is one of the most suitable and reliable techniques to create nanoporous or nanofibrous scaffolds for biomedical purposes. The biomimetic composition, osteoconductivity, and mechanical strength of organic/inorganic nanoparticles were used to create composite scaffolds. Bone structure varies from macro to nano scale. Therefore, nanocomposites primarily consist of nano- and micro-scale components that perform macro-scale functions at the tissue level. Further research focuses on designing composite nanofibrous scaffolds based on the nanofibrous morphology of native collagen. Calcium carbonates and bioactive glass nanoparticles can now be added to the list of potentially relevant mineral elements, previously confined to HAP and TCP found in natural bone tissue. Nanofibrous composite scaffolds can remain as the basis for BTE because of their biomimetic qualities. GFs or proteins may be loaded into biomimetic scaffolds to enhance cell adhesion, cell differentiation, and tissue regeneration. The mechanical strength of electrospun nanofibrous scaffolds must be enhanced for biomaterials scaffolds as structural support to match new tissue creation. The ideal alignment of nanofibers is of great importance to mimic the structure of native tissues. With the use of electrospun nanofibers, bioactive molecules could be delivered on-site without suffering any loss in their activities or structures. The high porosity of electrospun nanofibrous scaffolds allows for the controlled release of the loaded bioactive molecules in therapeutic doses. Although there are certain drawbacks to this method in clinical applications, including limited cellular penetration, high fiber density, and potential solvent/crosslinker toxicity, these issues can be resolved using techniques such as electrospinning polymers and cells, reducing the density of fibers and increasing the width of pores. Ultimately, the further development of electrospinning and electrospun nanofibrous scaffolds must address a number of issues, including the incompatibility of material degradation and bone development rates, the inability to provide numerous growth factors, and the dearth of a cohesive network of new bone and blood arteries. Due to their biomimetic nature, nanofibrous scaffolds may be the cornerstone as the BTE field aims to be able to totally and effectively repair bone defects. There is still considerable work ahead because full regeneration has not been achieved, despite the advances, biomimicry, and efficient drug delivery offered by electrospun nanofibers. The integration of a programmable delivery system into nanofiber-based regenerative scaffolds will be a future strategy for dealing with this specific problem, and this will open up fascinating new possibilities for tissue regeneration. Employing nanofiber potential for varied degrees of stiffness and drug loading will enable the creation of nanostructured scaffolds with appropriate spatial and temporal control. The integration and refinement of electrospinning technologies may be a game changer for biomaterial-based scaffold design and approaches to successful tissue regeneration.

As a result, future therapies for bone problems will need to take a comprehensive approach.

## Figures and Tables

**Table 1:** Recent in vitro and in vivo studies in BTR using electrospun nanofibrous scaffolds.

Biomaterial	Bioactive Agent	Cell-Line	Bone Defect	Assessment Parameters	Effect	Ref.
PLGA/PCL	Baicalin	BMSCs (bone mesenchymal stem cells)	5 mm	SEM, proliferation, cytotoxicity, RT-PCR, and polarization of RAW264.7 cells were performed to study surface morphology, cell viability, adhesion, and tissue regeneration	In vitro, scaffolds promoted osteogenic differentiation, and in vivo, scaffolds regulated inflammation and osteoclast differentiation and favored neovascularization and bone formation	[24]
PCL/Zein	Illite	MC3T3-E1	-	The WST-1 assay and ALP (alkaline phosphatase staining) was performed to study cell viability and osteoblastic differentiation	In vitro biomineralization of the scaffolds resulted in maximum calcium deposition (Ca/P ratio of 1.55), strong cell survival, and osteoblastic development	[25]
Collagen/PCL	Fe-dopped hydroxyapatite nanorods	MC3T3-E1	1 mm	Characterization, antioxidant potential, cytocompatibility, and aspects of osseointegration, including cell adhesion, proliferation, and bone formation, were studied	In an in vitro test, better-supported cell adhesion, cell growth, and matrix mineralization were reported, and in the in vivo study, the scaffold promoted osteointegration around bone–implant interface	[26]
Segmented polyurethane urea	Carboxyl carbon nanotube-doped hydroxyapatite	NIH3T3	3.5 mm	MTT, FESEM, and contact angle RT-PCR were performed to study morphology, cell viability, and adhesion	The in vitro test indicated excellent cytocompatibility and upregulated osteogenic gene expression. In vivo study showed excellent bone regeneration	[27]
PCL/PEG	Nano-attapulgite	D1 (mouse multipotent mesenchymal precursor)	5 mm	SEM, RT-PCR, and histological and immunohistochemical analyses were performed to study the biocompatibility of the scaffolds, osteogenesis, and new bone growth in rat cranium defect models	The in vitro study facilitated the differentiation of MSCs into osteoblasts and increased osteogenic gene expression. In vivo test exhibited an excellent bone regeneration effect and enhanced bone formation via the BMP/Smad signaling pathway	[28]
Silk fibroin	kappa-carrageena; k-CG)	MC3T3-E1	-	Characterizations, MTT bioassay, ALP, and confocal microscopy analysis were performed	Better cell viability and proliferation were observed, inducing mineralization and guiding MC3T3-E1 toward the osteogenic lineage	[29]

**Table 2:** List of some nanofibrous scaffold fabricated through electrospinning for bone regeneration.

Polymer	Solvent	Outcome	Ref.
HA/Col/CS (Poly(ethylene oxide): used as a sacrificial template)	Acetic acid, dimethyl sulfoxide (DMSO), and distilled water	The scaffolds demonstrated osteogenic differentiation	[164]
HA/Col (PVP: used as a sacrificial template)	Ethyl alcohol	The scaffolds beneficial to cell growth, proliferation, and material metabolism	[165]
SF/HA (PEO: used as sacrificial template)	Distilled water	In an in vitro test, the scaffolds improved osteogenic differentiation, and in an in vivo test, the scaffolds improved bone defect repair	[166]
Core-shell PLGA/PCL-BMP-2	2,2,2-Trifluoroethanol (TFEA), bovine serum albumin (BSA), distilled water	The scaffolds enhanced cell proliferation and osteogenic differentiation	[167]
CS/Polyamide 6,6	HFIP (Hexafluoro-2-isopropanol), and acetic acid	Cell growth, adhesion, differentiation, and proliferation were all improved as the concentration of CS was increased	[168]
PCL/Carboxymethyl chitosan	Formic acid:acetic acid (3:2, *v*/*v*)	The scaffolds enhanced cell proliferation and adhesion when compared with the PCL/chitosan scaffold	[128]
PCL/HA	Dichloromethane: N,N-dimethylformamide (3:2, *v*/*v*)	Biomimetic scaffold exploited osteoinduction, osteoconduction, and osteocompatibility in an in vitro study	[169]
PVA/PCL/HA-bioceramic	Chloroform:methanol (7:3, *v*/*v*) and distilled water	The scaffold promoted proliferation, osteoblastic differentiation, and evolution of stromal stem cells	[170]
SF/PCL	Formic acid	In in vitro research, the functionalized scaffolds improved cytocompatibility and osteogenic differentiation	[171]
SF/PLCL (poly(l-lactic acid-co-ε-caprolactone))	HFIP	In an in vivo study, the nanofibrous scaffold was found to be a suitable biomaterial for the tendon-bone mending	[172]
CS/PEO/silica hybrid nanofibers	Ethanol and acetic acid	The scaffold enhanced cytocompatibility, cell attachment, and proliferation	[173]
Alginate/HA	Acetic acid	No cytotoxicity, good biocompatibility and osteoconductivity	[174]
Poly (3-hydroxybutyrate)-CS/Alumina	Trifluoroacetic acid	The scaffold containing alumina had better MG-63 cell growth and feasibility, as well as the maximum alkaline phosphatase secretion than for the PHB or PHB/CS scaffolds	[175]
Cellulose	Acetone:water	Cell growth and proliferation	[176]

**Table 3:** List of nanofibrous scaffolds used in BTE for the release of drugs and GFs.

GF(s)	Carrier Material	Drugs	Application	Ref.
	Collagen PEG, PLGA, Polylactide/polyglicolide	Gentamicin Tobramycin and cefazolin gentamicin	Osteomyelitis	[224]
BMP-7	Poly(d,l-lactic acid) (PDLLA)	Pamidronate	Bone formation	[225]
-	HA-coated starch scaffold	Sodium clodronate	Bone tissue regeneration	[226]
BMP-2	Alginate/Collagen	-	Regeneration of femoral segmental defects	[227]
-	PCL loaded with HA and BP PLGA/HA	Clodronate Alendronate	Bone formation	[228]
PDGF-A	PLGA	-	Enhanced bone regeneration	[229]
BMP-2	Coating of silica xerogel–chitosan on porous HA scaffold	-	Improved bone regeneration	[230]
rhBMP2	SF/PLGA	DXM (dexamethasone)	Promising potential for boosting of bone tissue regeneration	[231]
TGF-β3 and BMP-2	PCL-POEGMA poly (oligoethyleneglycol methacrylate) scaffolds	-	Enhanced osteochondral differentiation of human mesenchymal stromal cells	[232]
VEGF	Polylactide + alginate	-	Decent VEGF discharge rate, boosted neovascularization in the bone healing process, and preserved bioactivity	[233]
-	Gel/HA	Ascorbic acid β-glycerophosphate disodium salt hydrate	Promoted osteoblast differentiation and induced bone regeneration	[234]
-	PCL/Gel/nanosilicate	Alendronate	Accelerated bone regeneration	[235]

## Data Availability

Not applicable.
